# A Method for Predicting Hemolytic Potency of Chemically Modified Peptides From Its Structure

**DOI:** 10.3389/fphar.2020.00054

**Published:** 2020-02-20

**Authors:** Vinod Kumar, Rajesh Kumar, Piyush Agrawal, Sumeet Patiyal, Gajendra P.S. Raghava

**Affiliations:** ^1^Department of Computational Biology, Indraprastha Institute of Information Technology, Okhla, India; ^2^Bioinformatics Centre, CSIR-Institute of Microbial Technology, Chandigarh, India

**Keywords:** modified hemolytic peptides, machine learning, chemical descriptors, fingerprints, random forest, HemoPImod

## Abstract

In the present study, a systematic effort has been made to predict the hemolytic potency of chemically modified peptides. All models have been trained, tested, and evaluated on a dataset that contains 583 modified hemolytic peptides and a balanced number of non-hemolytic peptides. Machine learning techniques have been used to build the classification models using an immense range of peptide features that include 2D, 3D descriptors, fingerprints, atom, and diatom compositions. Random Forest based model developed using fingerprints as an input feature achieved maximum accuracy of 78.33% with AUC of 0.86 on the main dataset and accuracy of 78.29% with AUC of 0.85 on the validation dataset. Models developed in this study have been incorporated in a web server “HemoPImod” to facilitate the scientific community (http://webs.iiitd.edu.in/raghava/hemopimod/).

## Introduction

**Figure 1:**
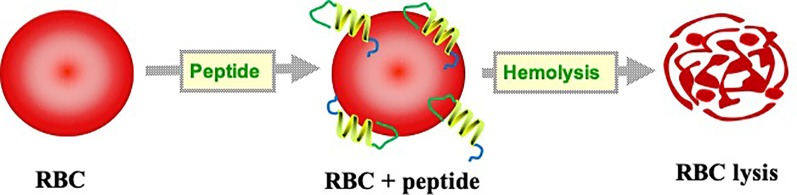
Figure illustrating mechanism of hemolysis by peptides.

Development of a new class of biologics and biologics-based drugs gains more importance in today's world. Among the biologics-based drugs, the peptide is a major class of molecule for the pharmaceutical companies as they are complacent of small molecules but biochemically and therapeutically different (Uhlig et al., 2014). The peptide-based therapeutics have a wide range of advantages over the conventional approach in terms of high target selectivity with minimum side effects (Oo and Kalbag, 2016). Apart from this, peptide based mimetics serve an attractive class to design new drug carriers, lead compounds, and excipients (Songok et al., 2018). Advancement in high-throughput screening and peptide synthesis techniques mark the avenue of peptide-based drug era. There are currently more than a hundred peptide-based drugs in the clinical trial development phases (Lau and Dunn, 2018). However, enthusiasm in peptide research is tempered by some intrinsic limitation of peptides such as immunogenicity (Fernandez et al., 2017), short half-life, proteolytic degradation, low bioavailability (Bruno et al., 2013), and toxicity (Chaudhary et al., 2016). Hemolytic concentration (HC_50_) is the commonly used indicator of peptide toxicity (Ruiz et al., 2014). HC_50_ refers to the 50% lysis of normal human erythrocytes under physiological conditions. Peptide rich in positively-charged amino acids binds to the negatively charged lipid bilayer of erythrocyte, leads to membrane disintegration and thus allowing water and other solute molecules to enter into the cell. This will increase the osmotic gradient inside the erythrocyte, which leads to cell swelling and bursting (Li et al., 2005) (**Figure 1**).

To improve the pharmacological properties of peptide-based drugs, a wide range of chemical and structural modifications have been proposed in the past. It includes PEGylated peptides (Kapoor et al., 2019), peptide lipidation (Menacho-Melgar et al., 2019), peptide acetylation and amidation (da Silva et al., 2014), incorporation of unnatural D-amino acids (Khara et al., 2016), and N-methylation (Chatterjee et al., 2008), etc. The overall goal of chemical/structural modification in the peptide is to improve solubility (Mahajan et al.), membrane permeation and decrease hemolysis (Lee and Lee, 2008) without tempering the therapeutic activity. In the past, numerous resources or databases have been developed to maintain different type of peptides that include cell-penetrating (Agrawal et al., 2016), antihypertensive (Kumar et al., 2015), anti-tuberculosis (Usmani et al., 2018), etc. properties. Besides, numerous tools have been developed to predict the therapeutic properties of natural peptides like ToxinPred for toxicity (Gupta et al., 2013), Antifp for antifungal (Agrawal et al., 2018), etc. Limited attempts have been made to predict the therapeutic properties of modified peptides that include ‘CellPPD-MOD' for modified cell-penetrating peptides (Kumar et al., 2018) and ‘AntiMPmod' for modified antimicrobial (Agrawal and Raghava, 2018) peptides. Although attempts have been made to predict the hemolytic potency of natural peptides (Raghava et al., 1994; Chaudhary et al., 2016; Win et al., 2017), thus far, there is no method that can predict the hemolytic potency of chemically modified peptides. The present study aims to develop various machine learning-based models to predict the hemolytic potency of chemically and structurally modified peptides.

## Materials and Methods

### Creation of Dataset

We extracted chemically modified hemolytic peptides from Hemolytik database (Gautam et al., 2014), which stores experimentally validated peptides with their hemolytic potencies. All the peptides satisfying the following criteria were selected for our datasets; i) Peptide has at least one modified amino acid; ii) Hazardous Concentration (HC_50_) or Half Maximum Effective Concentration (EC_50_) should be ≤100 μM; iii) Minimum Hemolytic Concentration (MHC) should be ≤250 μg/ml; and iv) >10% hemolytic activity up to 100 μM (Chaudhary et al., 2016). Peptides that do not meet the criteria mentioned above are considered as non-hemolytic in nature and serve the basis for the generation of datasets having non-hemolytic peptides. Finally, we got a dataset of 583 hemolytic and 583 non-hemolytic peptides, where each peptide has at least one non-natural or chemically modified amino acid. We used the PEPstrMOD script (Singh et al., 2015) for predicting the structure of each peptide in our dataset. The peptide length was kept 5–30 amino acids because the PEPstrMOD script has a limitation, i.e., length of the peptide. These structures were used for computing a wide range of descriptors.

### Evaluation of Models

The extracted peptide data were categorized into two datasets, i.e., main dataset and validation dataset. The main dataset constitutes the 0.8 part of the complete dataset (i.e., 466 modified hemolytic and 466 non-hemolytic peptides), and the remaining validation dataset contains 0.2 part of the dataset (i.e., 117 modified hemolytic and 117 non-hemolytic peptides). The peptides in both datasets were selected randomly to avoid bias.

We trained and tested the models using five-fold cross-validation technique on the main dataset. Five-fold cross-validation is a commonly used protocol where data is divided into five equal sets, where four sets are used for training, and the remaining set is used for validating the performance of the model. This process was iterated until each set was used once in testing the model. The final performance of the developed model was estimated by moderating the performance of each set (Nagpal et al., 2018). The external dataset is used to evaluate the overall performance of the best trained model developed on the main dataset.

### Model Development

#### Computation of Peptide Descriptors

Peptide descriptors or features such as atomic descriptors (atom composition, diatom composition), and chemical descriptors (2D, 3D, fingerprints, and combined) were utilized to develop various machine learning prediction models.

#### Atom Composition

To compute the atomic composition of modified hemolytic and non-hemolytic peptides, firstly, peptide tertiary structures were converted into SMILES (Simplified Molecular-input Line-entry System) format using Open Babel software. It is an open-source software that is routinely used in computational chemistry and other related areas to interconvert file formats (O'Boyle et al., 2011). The generated SMILE format of peptide structures was used to figure out the atomic composition of peptides viz. C, H, O, N, S, Cl, Br, and F. This led to the generation of a vector size of eight, and the formula used to compute this is as follows:-

(1) $$Percent\;composition\;of\;atom(a)=\;\frac{Total\;no.\;of\;atom(a)}{Total\;no.\;of\;all\;possible\;atoms}\times 100$$

Where atom (a) is a single atom from the above mentioned eight atoms.

#### Diatom Composition

The diatom composition of the peptides was computed in the same way as the composition of atoms. It helped to get a better understanding about the pair of atoms in every peptide, e.g., C-N, C-C, C-O, C-S, C-H, etc. The formula used to compute the diatom composition of peptide utilizes a vector size of 64 and is as follows:

(2) $$Percent\;composition\;\;of\;Diatom(a)=\;\frac{Total\;no.\;of\;Diatom(a)}{Total\;no.\;of\;all\;possible\;diatoms}\times 100$$

Where diatom (a) is a pair of atoms from possible 64 diatoms.

#### Chemical Descriptors

Structure-activity relationship (SAR) is often used in QSAR-based studies as there is a correlation between the molecular structure of a compound and its biological activity (Mafud et al., 2016). In the present study, we used PaDEL, an open-source software for calculating various descriptors of peptides (Yap, 2011). We used this software for calculating 2D, 3D descriptors, and fingerprints.

#### Feature Selection

It has been shown in the past that all descriptors do not correlate with biological activity. Hence, we removed unnecessary descriptors as they can create noise in data and may lead to false repercussions. To remove such bias, while developing the prediction model, we used a feature selection technique using WEKA, an open-source software, at its default parameters (Smith and Frank, 2016). We applied “CfsSubsetEval” as an attribute evaluator with “BestFirst” as a search method in WEKA software with default settings in the forward direction with lookup size, D  =  1, and amount of backtracking, N  =  5.

#### Machine Learning Methods

To predict the nature of chemically modified hemolytic peptide, we employed different machine learning algorithms using Scikit-learn. We implemented widely used classifiers as described below, along with their default parameters. 1). Ridge classifier: It classifies the data by using parameters that include alpha, max iter, and solver that controls the processing of classifiers. The classifier learns the model and generates a coefficient vector that best fits the data (Grüning and Kropf, 2006). 2). Random forest (RF): This is a tree-based classifier algorithm, which trains each decision tree with the different training datasets. The new object is classified based on the votes given by each tree in the forest for the attributes of the new object (Robu and Hora, 2012). 3). K-nearest neighbor (KNN): This Method classifies the new object based on the distance to the labelled/known instances in the training dataset (Hussain et al., 2015). 4). Extra Tree: Predicts the outcome of the new object by taking the average of outputs from all aggregated trees (Geurts et al., 2006).

#### Performance Measure

The outcome of the generated model was assessed using various parameters that are threshold-dependent and threshold-independent. The threshold-dependent parameters used in this study are sensitivity (Sen), Specificity (Spc), Accuracy (Acc), and Matthews correlation coefficient (MCC), using the following equations. These measurements obtained from these parameters are expressed in terms of true positive (TP), false negative (FN), true negative (TN), and false positive (FP).These can be calculated using equations 3–6.

(3) $$Sensitivity=\;\frac{TP}{TP+FN}\times 100$$

(4) $$Specificity=\;\frac{TN}{TN+FP}\times 100$$

(5) $$Accuracy=\;\frac{TP+TN}{TP+FP+TN+FN}\times 100$$

(6) $$Matthew^{\prime }s\;Correlation\;Coefficient=\;\frac{\left(TN\times TP)-(FN\times FP\right)}{\sqrt{\text{(FP+TP)(FN+TP)(FP+TN)(FN+TN)}}}\times 100$$

Where TN and TP denote perfectly predicted modified non-hemolytic peptides and hemolytic peptides, respectively. FN and FP denote badly predicted modified non-hemolytic peptides and hemolytic peptides, respectively.

Most of the above-used measurements have a drawback—the performance of the developed models depends on the threshold. To overcome this bias, we adopted threshold-independent parameters to evaluate the performance of developed models. A well-known threshold-independent measure is Receiver Operating Characteristics (ROC). We computed the area under curve (AUC) in ROC plot to get the overall performance. pROC package developed in R was used for computing the AUROC (Robin et al., 2011).

## Results

We used Scikit-learn (a Python library) for developing prediction models by employing diverse approaches of machine learning like Ridge Classifier, Random Forest, KNN, and ExtraTree. The developed models were based on different features/descriptors, which can discriminate modified hemolytic peptides from non-hemolytic ones. The interpretation of results is provided below in detail.

### Structure-Based Model

To develop the structure-based model, the tertiary structure of hemolytic peptides is generated using PEPstrMod (Singh et al., 2015). These structures were further used for extracting different types of features and descriptors. The model is created using discrete structural features of the peptide. First, the model is developed by using the atomic composition of peptide tertiary structures. To compute the atomic composition of peptide, structure data format (sdf) is first converted to SMILES, and then the atomic composition was computed. Prediction models were developed using the Scikit-learn library by implementing different classifiers like ExtraTree, RF, KNN, and Ridge classifier using an input feature as atomic composition. RF-based classification ML model yielded the highest accuracy, which is 70.49%, MCC of 0.41, and AUC of 0.81 on the main dataset. The performance attained on the validation dataset has 69.66% accuracy, MCC of 0.39, and AUC of 0.78. Performance of various methods with parameters are presented in **Table 1**.

**Table 1:** Performance achieved by scikit ML on the composition of the atoms.

Methods (Parameters)	Main Dataset					Validation Dataset				
	Sen	Spc	Acc	MCC	AUC	Sen	Spc	Acc	MCC	AUC
RF (n_estimators = 100)	72.75	68.24	70.49	0.41	0.81	68.89	70.43	69.66	0.39	0.78
KNN (n_neighbors = 5,algorithm = ‘brute',weights = ‘distance')	70.39	68.24	69.31	0.39	0.79	68.38	71.79	70.09	0.4	0.80
Ridge (alpha = 0.01)	54.51	59.23	56.87	0.14	0.72	52.99	58.12	55.56	0.11	0.71
Extratree (n_estimators = 60)	74.03	66.74	70.39	0.41	0.81	74.87	68.03	71.45	0.43	0.82

Beside atomic composition, the diatomic composition-based model was also developed. The model achieves the highest accuracy of 74.36% with MCC of 0.49 and AUC of 0.87. On the validation dataset, we gathered the accuracy of 75.98% with MCC 0.52 and AUC of 0.88. Here, the ExtraTree-based model performed best among all the classifiers used for prediction. Performance of various methods with parameters are presented in **Table 2**.

**Table 2:** Performance achieved by scikit ML on the composition of the diatom.

Methods (Parameters)	Main Dataset					Validation Dataset				
	Sen	Spc	Acc	MCC	AUC	Sen	Spc	Acc	MCC	AUC
RF (n_estimators = 100)	73.61	74.03	73.82	0.48	0.83	78.46	74.36	76.41	0.53	0.86
KNN (n_neighbors = 10,algorithm = ‘ball_tree',weights = ‘uniform')	72.32	61.59	66.95	0.34	0.81	73.5	72.65	73.08	0.46	0.84
Ridge (alpha = 1)	57.51	57.51	57.51	0.15	0.72	55.56	63.25	59.4	0.19	0.75
Extratree (n_estimators = 200)	75.54	73.18	74.36	0.49	0.87	77.78	74.19	75.98	0.52	0.88

### Chemical Descriptors-Based Prediction

We used PaDEL software to compute 2D descriptors, 3D descriptors, and fingerprints from the tertiary structure of peptides. We then used WEKA to select the best features using “CfsSubsetEval” with the search method of “BestFirst” at default parameters, as explained in the *Materials and Methods* section. Individual models for 2D descriptors, 3D descriptors, and Fingerprints, as well as a single model combining all descriptors, were developed. In the case of 2D descriptors, a total of 221 descriptors were calculated initially, and then 20 features were selected by implementing the feature selection technique. We applied different machine learning techniques on both the datasets, i.e., before and after feature selection, and observed that the RF-based model achieved the maximum accuracy of 75.88%, MCC of 0.52, and AUC of 0.83 for the main dataset and 76.21% accuracy, 0.52 MCC, and 0.81 AUC for the validation dataset before feature selection (**Table 3**). But in the case of the dataset after feature selection, ExtraTree-based model achieved the maximum accuracy of 75.66%, MCC of 0.51, and AUC of 0.82 for the main dataset and 74.54% accuracy, 0.49 MCC, and 0.80 AUC for the validation dataset (**Table S1**).

**Table 3:** Performance achieved by scikit ML on the 2D descriptors.

Methods (Parameters)	Main Dataset					Validation Dataset				
	Sen	Spc	Acc	MCC	AUC	Sen	Spc	Acc	MCC	AUC
RF (n_estimators = 1000)	79.37	72.49	75.88	0.52	0.83	76.76	75.69	76.21	0.52	0.81
KNN (n_neighbors = 8,algorithm = ‘kd_tree',weights = ‘distance')	67.94	61.35	64.6	0.29	0.72	61.26	63.79	62.56	0.25	0.67
Ridge (alpha = 1)	71.08	78.38	74.78	0.5	0.81	58.56	82.76	70.93	0.43	0.74
Extratree (n_estimator = 40)	76.01	73.8	74.89	0.5	0.82	73.87	77.07	75.51	0.51	0.80

In the case of 3D descriptors, a total of 20 features were calculated and were reduced to 4 after applying feature selection. Of the 20 features, ExtraTree model performed better than other models and achieved maximum accuracy of 65.74%, MCC of 0.31, and AUC value of 0.70 on the main dataset and 63.25% accuracy, 0.27 MCC, and 0.68 AUC on the validation dataset (**Table 4**). But of the 4 reduced features, the RF model performed better than other models and achieved maximum accuracy of 63.59%, MCC of 0.27, and AUC value of 0.69 on the main dataset and 61.97% accuracy, 0.24 MCC, and 0.67 AUC on the validation dataset (**Table S2**).

**Table 4:** Performance achieved by scikit ML on the 3D descriptors.

Methods (Parameters)	Main Dataset					Validation Dataset				
	Sen	Spc	Acc	MCC	AUC	Sen	Spc	Acc	MCC	AUC
RF (n_estimators = 800)	60.22	66.09	63.16	0.26	0.69	58.29	65.64	61.97	0.24	0.67
KNN (n_neighbors = 10,algorithm = ‘ball_tree',weights = ‘distance')	60.43	54.94	57.68	0.15	0.61	51.28	58.97	55.13	0.1	0.59
Ridge (alpha = 0.01)	58.71	60.73	59.72	0.19	0.65	49.57	63.25	56.41	0.13	0.59
Extratree (n_estimator = 70)	65.59	65.88	65.74	0.31	0.70	61.71	64.79	63.25	0.27	0.68

The different types of fingerprints generated 13,508 features, which were reduced to 28 after feature selection. The performance of different classifiers was evaluated on 13,508 features (**Table 5**), and RF showed the best performance with a maximum accuracy of 78.33%, MCC of 0.56, and AUC of 0.86 on the main dataset and accuracy of 78.29%, MCC of 0.57, and AUC of 0.85 on the validation dataset. In the case of the 28 reduced features, RF showed the best performance with an accuracy of 78.31%, MCC of 0.57, and AUC of 0.86 on the main dataset and accuracy of 75.56%, MCC of 0.51, and AUC of 0.83 on the validation dataset (**Table S3**).

**Table 5:** Performance achieved by scikit ML on the fingerprints descriptors.

Methods (Parameters)	Main Dataset					Validation Dataset				
	Sen	Spc	Acc	MCC	AUC	Sen	Spc	Acc	MCC	AUC
RF (n_estimators = 800)	77.51	77.16	78.33	0.56	0.86	80.85	75.73	78.29	0.57	0.85
KNN (n_neighbors = 8,algorithm = ‘ball_tree',weights = ‘distance')	75.98	70.69	73.32	0.47	0.81	77.78	70.94	74.36	0.49	0.79
Ridge (alpha = 1)	75.76	71.55	73.64	0.47	0.81	76.92	70.09	73.5	0.47	0.80
Extratree (n_estimator = 300)	78.6	73.71	76.14	0.52	0.84	78.46	75.56	77.01	0.54	0.82

Finally, we combined all the 2D, 3D descriptors, and fingerprints at the same time, which generated 13,739 features. Feature selection on all combined descriptors leads to 34 features. Of the 13,739 features, we observed the maximum accuracy of 78.42%, MCC of 0.57, and AUC of 0.86 on the main dataset and 78.46% accuracy, 0.57 MCC, and 0.84 AUC on the validation dataset by RF model (**Table 6**). In the case of the 34 reduced features, Extratree showed a maximum accuracy of 77.9%, MCC of 0.56, and AUC of 0.85 on the main dataset and accuracy of 74.44%, MCC of 0.49, and AUC of 0.81 on the validation dataset (**Table S4**).

**Table 6:** Performance achieved by scikit ML on the 2D, 3D, and fingerprints descriptors.

Methods (Parameters)	Main Dataset					Validation Dataset				
	Sen	Spc	Acc	MCC	AUC	Sen	Spc	Acc	MCC	AUC
RF (n_estimators = 200)	77.73	79.09	78.42	0.57	0.86	79.83	77.09	78.46	0.57	0.84
KNN (n_neighbors = 10,algorithm = ‘kd_tree',weights = ‘distance')	62.88	62.5	62.69	0.25	0.67	49.57	58.97	54.27	0.09	0.60
Ridge (alpha = 1)	62.45	53.02	57.7	0.16	0.61	63.25	48.72	55.98	0.12	0.58
Extratree (n_estimator = 1000)	80.35	74.35	77.33	0.55	0.85	82.05	72.31	77.18	0.55	0.83

**Figure 2:**
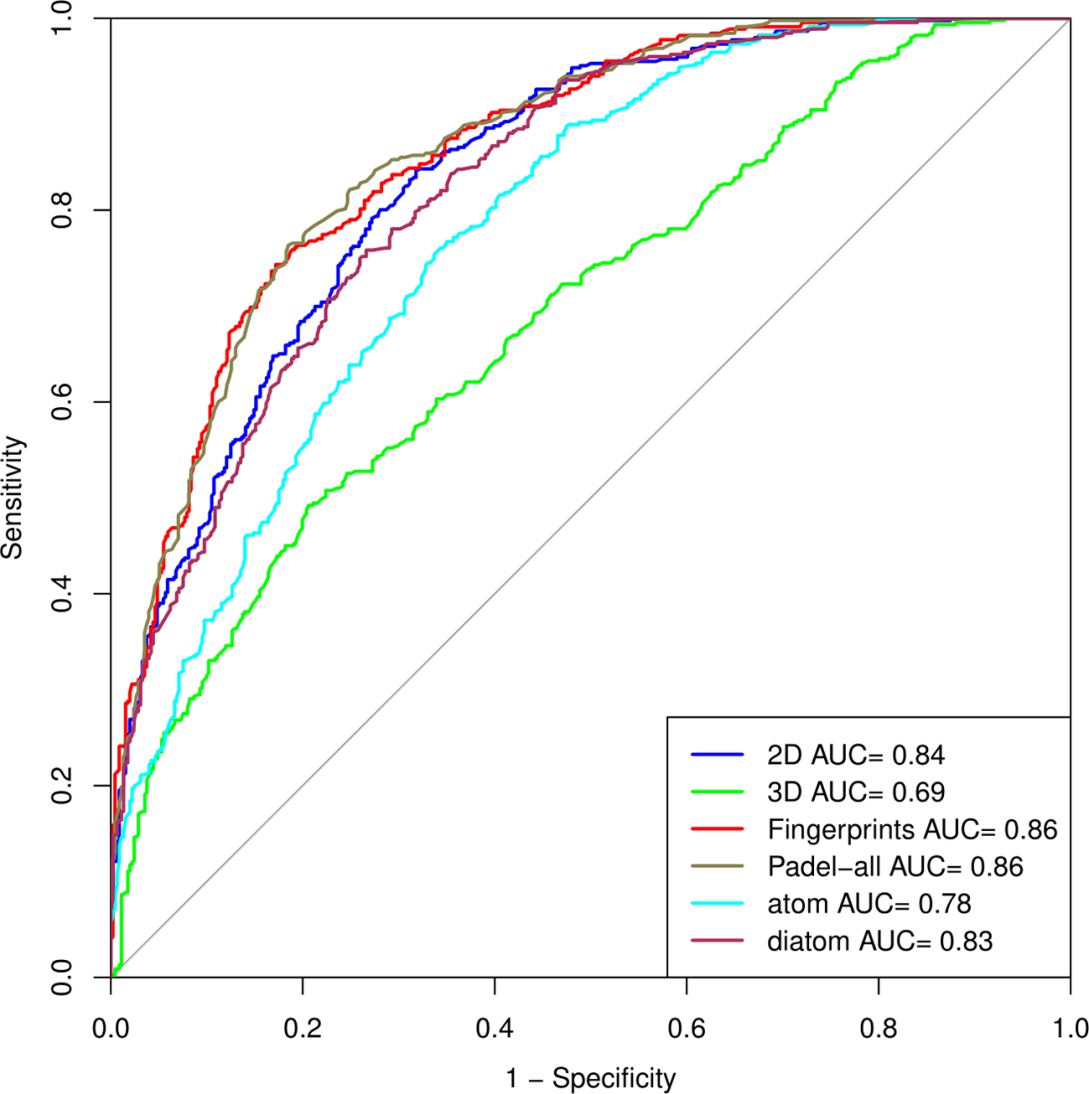
Outcome of the model on various structural features as a ROC curve.

We prepared the ROC curve (Robin et al., 2011) of all the datasets, i.e., 2D, 3D, fingerprints, and the combination of three, (all PaDEL descriptors), atom composition, and diatom composition (**Figure 2**), to compare the performances of models on various structural features.

### Webserver Implementation

HemoPImod (https://webs.iiitd.edu.in/raghava/hemopimod/) is developed as a computational tool to facilitate the scientific community. The RF-based model performed best among all the models, hence implemented in the webserver. This model helps to predict the hemolytic or non-hemolytic potential of the modified peptide. The user interface of the tools is deliberately kept very simple. The only required input is the tertiary structure of the modified peptide in the PDB format. If a user doesn't have the tertiary structure of the modified peptide, the structure can be generated from PEPstrMOD. From the threshold panel, the user is advised to select the appropriate threshold value. After data processing, the result page provides information on the nature of input peptide with probability score, and in the text, as well as graphical form. We are also providing a standalone version of the model, which is present in the webserver and integrated into the GPSRdocker (Agrawal et al., 2019).

## Discussion

In the last few decades, emphasis on the development of the therapeutic peptides has been increased. Most of the clinically approved therapeutic peptide drugs act as a natural substance in the human body. Therapeutic peptides have various limitations, such as short half-life, oral bioavailability, etc., which decreases their efficacy. These kinds of limitations can be improved with the help of modifications in the peptide (Bruno et al., 2013) such as chemical modification in some CPPs, which improved its bioavailability like cysteine residue modification enhanced the stability of Tat peptide and thus enhanced the plasmid delivery (Lo and Wang, 2008). Polyethylene glycol (PEG), lipids, and proteins such as Fc fragments has been used as a half-life extension strategy (Wang and Ying, 2016). Hence, modification is an important aspect of peptide-based therapeutic drug development. Thus, various computational research is being focused on modified peptides such as “Prediction of Cell-Penetrating Potential of Modified Peptides Containing Natural and Chemically Modified Residues” (Gautam et al., 2013; Gautam et al., 2015; Kumar et al., 2018), “Antimicrobial Potential of a Chemically Modified Peptide” (Agrawal and Raghava, 2018), etc. While developing therapeutic peptides, consideration of its hemolytic activity is an important step. In the past, various computational methods were developed that are capable of predicting the hemolytic potency of the peptides [for instance, Hemopi (Chaudhary et al., 2016)], but all of them were based on peptides possessing only natural amino acids. But as technology permits, modifications can be considered as features in computational methods by considering the structural information. Hence, by keeping the importance of peptide modification in mind, we have developed a computational method to predict the hemolytic activity of peptides based on the structural features. We developed the computational method with the help of various machine learning techniques such as RF, KNN, Ridge, and ExtraTree by using different kind of datasets such as atom composition, diatom composition, PaDEL descriptors (2D, 3D, and fingerprints) as well as by combining 2D, 3D descriptors, and fingerprints.

We obtained the best performance by implementing RF using PaDEL descriptors (fingerprints) as input feature with an accuracy of 78.33%, MCC of 0.56, and AUC of 0.86 on the main dataset and accuracy of 78.29%, MCC of 0.57, and AUC of 0.85 on the validation dataset. We hold an opinion that the development of this method will highly assist in the research of therapeutic peptides-based drug development. As better tools for structure prediction will develop, we will be able to improve this computational method; due to shortcomings of the structure prediction tools, we are not able to incorporate peptides beyond the length of 7–25 amino acid and with modifications that are present in PEPstrMOD. Hence, in conclusion, this method can be improved by the improvement in the structure prediction tool.

## Data Availability Statement

The datasets generated for this study can be found at https://webs.iiitd.edu.in/raghava/hemopimod/download.php.

## Author Contributions

VK generated the dataset. VK, PA, and SP performed the experiments. VK performed data analysis and prepared the tables and figures. VK, RK, and SP developed the web interface. VK, RK, and GR wrote the manuscript. GR conceived the idea and coordinated the project.

## Conflict of Interest

The authors declared that the research was conducted in the absence of any commercial or financial relationships that could be construed as a potential conflict of interest.
