# Supplementary material for: A Method for Predicting Hemolytic Potency of Chemically Modified Peptides From Its Structure
Source: Front Pharmacol. 2020 Feb 20;11:54. doi: 10.3389/fphar.2020.00054 (PMC7045810; doi:10.3389/fphar.2020.00054)
Supplement: Supplementary file 1 [file DataSheet_1.docx]

**A Method for Predicting Hemolytic Potency of Chemically Modified Peptides from its Structure**

**Vinod Kumar^1,2^, Rajesh Kumar^1,2^, Piyush Agrawal^1,2^, Sumeet Patiyal^1^,*Gajendra P.S. Raghava^1^**

^1^Department of Computational Biology, Indraprastha Institute of Information Technology, Okhla, India

^2^Bioinformatics Centre, CSIR-Institute of Microbial Technology, Sector-39A, Chandigarh, India

* Corresponding author

Professor, Department of Computational Biology, Indraprastha Institute of Information

Technology, Okhla Industrial Estate, Phase III, New Delhi 110020.

India. Tel.: +91 011 26907444

E-mail address: raghava@iiitd.ac.in

Table S1: Performance of Scikit machine learning After Feature selection on the 2D descriptors

|  | Main Dataset | | | | | Validation Dataset | | | | |
| --- | --- | --- | --- | --- | --- | --- | --- | --- | --- | --- |
| Methods (Parameters) | Sen | Spc | Acc | MCC | AUC | Sen | Spc | Acc | MCC | AUC |
| RF (n_estimators=50) | 76.46 | 70.96 | 73.67 | 0.47 | 0.82 | 76.58 | 72.07 | 74.27 | 0.49 | 0.81 |
| KNN (n_neighbors=10,algorithm='ball_tree',weights='distance') | 69.73 | 69.43 | 69.58 | 0.39 | 0.77 | 67.57 | 65.52 | 66.52 | 0.33 | 0.72 |
| Ridge (alpha=0.1) | 69.73 | 70.09 | 69.91 | 0.4 | 0.77 | 63.06 | 70.69 | 66.96 | 0.34 | 0.72 |
| Extratree (n_estimator=40) | 78.7 | 72.71 | 75.66 | 0.51 | 0.82 | 77.84 | 71.38 | 74.54 | 0.49 | 0.80 |

Table S2: Performance of Scikit machine learning Results After Feature selection on the 3D descriptors

|  | Main Dataset | | | | | Validation Dataset | | | | |
| --- | --- | --- | --- | --- | --- | --- | --- | --- | --- | --- |
| Methods (Parameters) | Sen | Spc | Acc | MCC | AUC | Sen | Spc | Acc | MCC | AUC |
| RF (n_estimators=500) | 63.01 | 64.16 | 63.59 | 0.27 | 0.69 | 60 | 63.93 | 61.97 | 0.24 | 0.67 |
| KNN (n_neighbors=10,algorithm='kd_tree',weights='uniform') | 67.53 | 58.37 | 62.94 | 0.26 | 0.66 | 60.43 | 54.73 | 52.32 | 0.18 | 0.61 |
| Ridge (alpha=1) | 59.14 | 59.44 | 59.29 | 0.19 | 0.64 | 47.01 | 60.68 | 53.85 | 0.08 | 0.57 |
| Extratree (n_estimator=400) | 61.72 | 65.24 | 63.48 | 0.27 | 0.69 | 58.8 | 62.05 | 60.43 | 0.21 | 0.67 |

Table S3: Performance of Scikit machine learning Results After Feature selection on the fingerprints descriptors

|  | Main Dataset | | | | | Validation Dataset | | | | |
| --- | --- | --- | --- | --- | --- | --- | --- | --- | --- | --- |
| Methods (Parameters) | Sen | Spc | Acc | MCC | AUC | Sen | Spc | Acc | MCC | AUC |
| RF (n_estimators=600) | 78.6 | 78.02 | 78.31 | 0.57 | 0.86 | 75.73 | 75.38 | 75.56 | 0.51 | 0.83 |
| KNN (n_neighbors=5,algorithm='ball_tree',weights='distance') | 74.45 | 72.41 | 73.43 | 0.47 | 0.80 | 71.79 | 68.38 | 70.09 | 0.4 | 0.75 |
| Ridge (alpha=0.01) | 75.11 | 70.69 | 72.89 | 0.46 | 0.81 | 70.94 | 76.07 | 73.5 | 0.47 | 0.76 |
| Extratree (n_estimator=60) | 80.13 | 77.16 | 78.63 | 0.57 | 0.85 | 75.73 | 71.79 | 73.76 | 0.48 | 0.81 |

Table S4: Performance of Scikit machine learning Results After Feature selection on the 2D, 3D and fingerprints descriptors

|  | Main Dataset | | | | | Validation Dataset | | | | |
| --- | --- | --- | --- | --- | --- | --- | --- | --- | --- | --- |
| Methods (Parameters) | Sen | Spc | Acc | MCC | AUC | Sen | Spc | Acc | MCC | AUC |
| RF (n_estimators=800) | 74.95 | 75.86 | 75.41 | 0.51 | 0.85 | 75.21 | 76.58 | 75.9 | 0.52 | 0.83 |
| KNN (n_neighbors=10,algorithm='auto',weights='distance') | 67.76 | 64.87 | 66.31 | 0.33 | 0.71 | 65.81 | 63.25 | 64.53 | 0.29 | 0.65 |
| Ridge (alpha=0) | 76.69 | 70.69 | 73.67 | 0.47 | 0.82 | 73.5 | 73.5 | 73.5 | 0.47 | 0.76 |
| Extratree (n_estimator=300) | 80.61 | 75.22 | 77.9 | 0.56 | 0.85 | 78.46 | 70.43 | 74.44 | 0.49 | 0.81 |
